# Vangl-dependent planar cell polarity signalling is not required for neural crest migration in mammals

**DOI:** 10.1242/dev.111427

**Published:** 2014-08

**Authors:** Sophie E. Pryor, Valentina Massa, Dawn Savery, Philipp Andre, Yingzi Yang, Nicholas D. E. Greene, Andrew J. Copp

**Affiliations:** 1 Newlife Birth Defects Research Centre, Institute of Child Health, University College London, 30 Guilford Street, London, WC1N 1EH, UK; 2 Genetic Disease Research Branch, National Human Genome Research Institute, 49 Convent Drive, MSC 4472, Bethesda, MD 20892, USA

**Keywords:** Cell migration, Embryo, Mouse, Neural crest, Neural tube, Planar cell polarity

## Abstract

The role of planar cell polarity (PCP) signalling in neural crest (NC) development is unclear. The PCP dependence of NC cell migration has been reported in *Xenopus* and zebrafish, but NC migration has not been studied in mammalian PCP mutants. *Vangl2^Lp/Lp^* mouse embryos lack PCP signalling and undergo almost complete failure of neural tube closure. Here we show, however, that NC specification, migration and derivative formation occur normally in *Vangl2^Lp/Lp^* embryos. The gene family member *Vangl1* was not expressed in NC nor ectopically expressed in *Vangl2^Lp/Lp^* embryos, and doubly homozygous *Vangl1*/*Vangl2* mutants exhibited normal NC migration. Acute downregulation of *Vangl2* in the NC lineage did not prevent NC migration. *In vitro*, *Vangl2^Lp/Lp^* neural tube explants generated emigrating NC cells, as in wild type. Hence, PCP signalling is not essential for NC migration in mammals, in contrast to its essential role in neural tube closure. PCP mutations are thus unlikely to mediate NC-related birth defects in humans.

## INTRODUCTION

The neural crest (NC) is a transient cell population that delaminates from the dorsal neural tube and migrates extensively, generating a variety of cell types (Kulesa et al., 2010; Sauka-Spengler and Bronner-Fraser, 2008). NC emigration is closely coordinated spatiotemporally with closure of the neural tube, and some genes [e.g. *AP2α* (*Tfap2a*), *Cecr2*, *Pax3*, *Zic2*] (Harris and Juriloff, 2007) are necessary for both embryonic events. Signalling via the planar cell polarity (PCP) pathway is required for neural tube closure in vertebrates, and recently PCP mutations were reported in human neural tube defects (Juriloff and Harris, 2012). However, the role of PCP signalling in NC migration, particularly in mammals, remains unresolved.

The PCP pathway is an evolutionarily conserved, non-canonical Wnt-frizzled-dishevelled signalling cascade. The vertebrate homologues of *Drosophila* ‘core’ PCP genes regulate many developmental processes, including convergent extension (CE) cell movements in embryonic axis elongation, orientation of mechanosensory hair cells in the cochlea, and the arrangement of fur, feathers and scales (Seifert and Mlodzik, 2007).

In *Xenopus* embryos, disruption of PCP signalling (*Dsh*-DEP^+^ or dominant-negative *Wnt11* mRNA) inhibited cranial NC migration *in vivo* and *in vitro* (De Calisto et al., 2005). Similar findings were reported with the PCP-associated gene *PTK7* (Shnitsar and Borchers, 2008), and NC migration defects were also observed in zebrafish treated with *Dsh*-DEP^+^ or *wnt5* morpholino (Matthews et al., 2008). Knockdown of Strabismus (Vangl2 orthologue) inhibited *Xenopus* NC migration similarly to *Dsh*-DEP^+^ (Carmona-Fontaine et al., 2008), whereas a milder NC migration phenotype was observed in the *trilobite* (*vangl2*) zebrafish mutant (Matthews et al., 2008).

It is unclear whether PCP signalling is essential for mammalian NC migration. NC-related anomalies comprise up to 20% of clinically important human birth defects (Bolande, 1974; Dolk et al., 2010), so it is important to ascertain whether PCP mutations are a likely cause. Here, we examined NC migration in mice lacking Vangl1/2 function. *Loop-tail* (*Lp*) is a dominant-negative allele of the core PCP gene *Vangl2* that abrogates PCP signalling (Kibar et al., 2001; Murdoch et al., 2001; Song et al., 2010; Yin et al., 2012). *Vangl2^Lp^* homozygotes fail almost completely in neural tube closure due to defective CE in midline neural plate and axial mesoderm (Ybot-Gonzalez et al., 2007). They also display defects of cochlea organisation, heart morphogenesis, lung and kidney branching and reproductive system development – all attributed to severely disrupted PCP function (Montcouquiol et al., 2003; Torban et al., 2007; vandenBerg and Sassoon, 2009; Yates et al., 2010a, b). We find no defects in NC migration in *Vangl1/2* mutant embryos, either *in vivo* or *in vitro*, arguing strongly that PCP signalling is not essential for early NC development in mammals.

## RESULTS

### NC specification and migration are normal in *Vangl2^Lp/Lp^* embryos

The specification of NC cells was detected by whole-mount *in situ* hybridisation (WISH) for *Sox9*, a marker of premigratory NC (Cheung and Briscoe, 2003). *Sox9*-positive NC cells were visible along the mid-dorsal aspect of the embryonic day (E) 9.5 wild-type neural tube and, similarly, on the tips of the open neural folds in stage-matched *Vangl2^Lp/Lp^* embryos (supplementary material Fig. S1A-F).

**Fig. 1.**
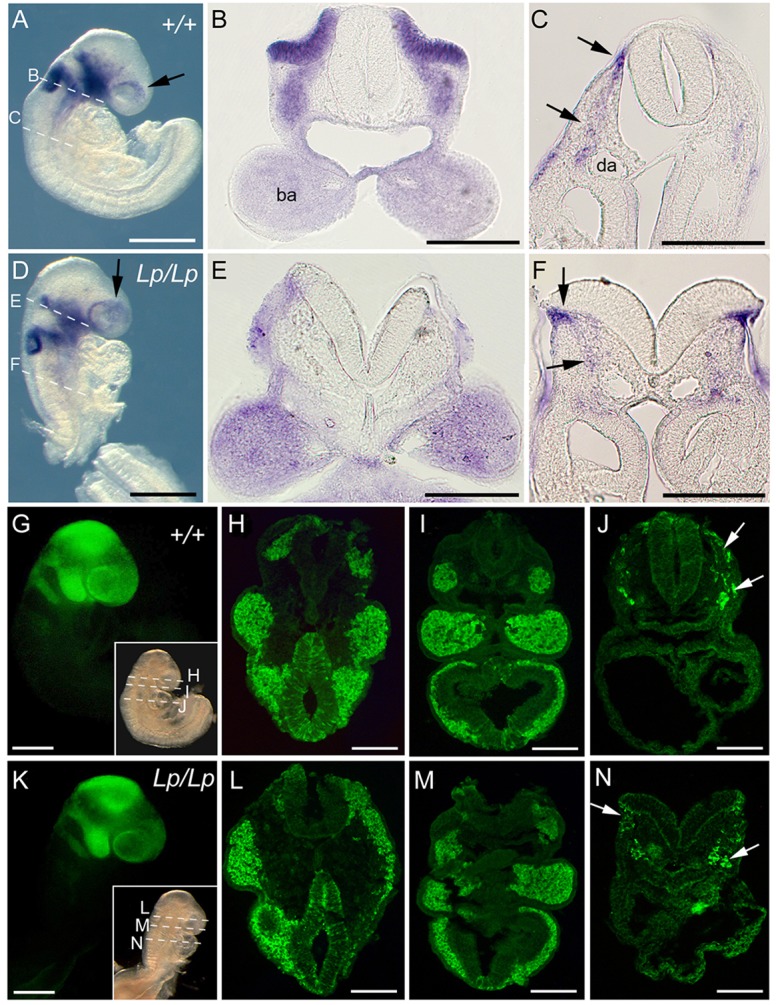
**Normal pattern of NC cell migration in *Vangl2^Lp/Lp^* mouse embryos.** Migrating NC detected by *Erbb3* mRNA expression (A-F) and YFP expression regulated by *Wnt1-Cre* (G-N). Wild-type (*+/+*; A-C,G-J) and *Vangl2^Lp/Lp^* (*Lp/Lp*; D-F,K-N) embryos at early E9.5 (13-14 somites) both exhibit NC cells colonising forebrain, peri-ocular region (A,D, arrows) and upper branchial arches (ba). Transverse sections show branchial arch colonisation (B,E,H,I,L,M) and migration from closed (+/+) and open (*Lp/Lp*) neural tube (arrows in C,F,J,N). da, dorsal aorta. Scale bars: 500 µm in A,D; 200 µm in B,C,E,F; 250 µm in G,K; 100 µm in H-J,L-N.

Migrating NC cells were detected by WISH for *Erbb3*, a neuregulin receptor tyrosine kinase (Garratt et al., 2000). Both wild-type and stage-matched *Vangl2^Lp/Lp^* embryos at E9.5 showed streams of cranial NC cells migrating from the hindbrain towards branchial arches 1 and 2, and around the optic vesicles (Fig. 1A,B,D,E). *Erbb3*-positive trunk NC cells were delaminating from the neuroepithelium and migrating ventrally (Fig. 1C,F). Later in development, NC cell emigration from the trunk neural tube also appeared closely comparable in wild-type and *Vangl2^Lp/Lp^* embryos (supplementary material Fig. S1G-T).

A similar NC migration pattern was detected by fluorescent lineage labelling in both *Vangl2^+/+^;* *Wnt1-Cre/YFP* and *Vangl2^Lp/Lp^;* *Wnt1-Cre/YFP* embryos. At E8.5, YFP-positive NC cells had colonised the forebrain, peri-ocular region and branchial arches 1 and 2 (Fig. 1G-I,K-M), and migrating NC cells were present at heart-level in both genotypes (Fig. 1J,N). Closely comparable patterns of NC cell distribution were present later in development at different axial levels (supplementary material Fig. S2A-H). No significant differences were found in the number of migrating YFP-positive NC cells in *Vangl2^+/+^* and *Vangl2^Lp/Lp^* embryos at E9, E9.5 or E10.5 (supplementary material Fig. S2I). Analysis of embryos at E10.5, both by *Erbb3* WISH (supplementary material Fig. S1U-BB) and Wnt1-Cre/YFP labelling (supplementary material Fig. S2J-Y), also revealed very similar NC cell distribution and patterning of NC-derived structures. We conclude that specification, migration and tissue colonisation by NC is normal in *Vangl2^Lp/Lp^* mutants that fail in neural tube closure.

### Vangl1 does not compensate for loss of Vangl2 during NC migration

We examined whether the gene family member *Vangl1* could compensate for loss of *Vangl2*, thereby ensuring normal NC migration. *Vangl1* is a highly conserved, structurally similar paralogue of *Vangl2* (Torban et al., 2004) and the only other known mammalian orthologue of *Drosophila* Strabismus (Van Gogh)*.* Both Vangl1 and Vangl2 proteins interact physically with mammalian dishevelled (Torban et al., 2004). Moreover, *Vangl1* interacts genetically with *Vangl2* during neurulation (Torban et al., 2008), with a more severe phenotype in *Vangl1*/*Vangl2* double homozygotes than in *Vangl2^Lp/Lp^* (Song et al., 2010).

**Fig. 2.**
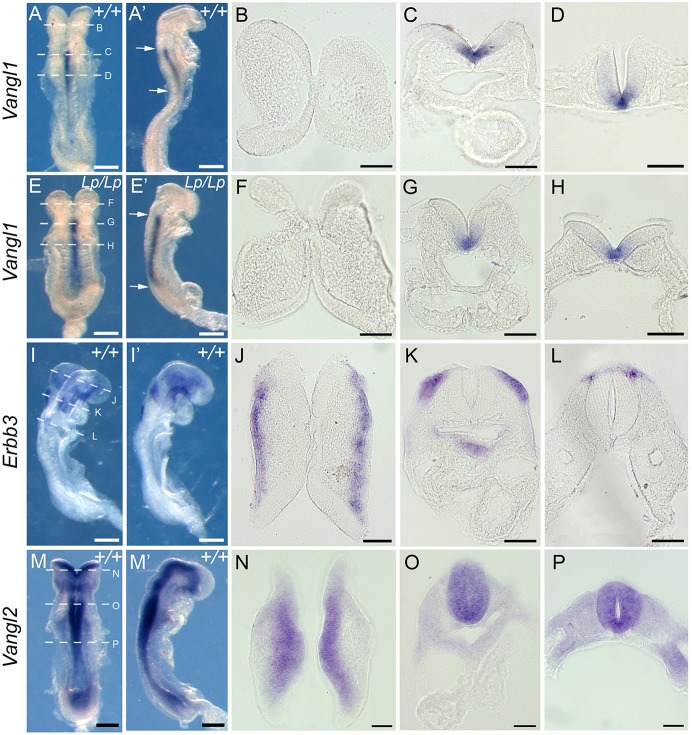
***Vangl1* is not expressed in wild-type NC, nor ectopically in *Vangl2^Lp/Lp^* mutants.** WISH in intact embryos shown from dorsal (A,E,I,M) and right lateral (A′,E′,I′,M′) views, and in sections (B-D,F-H,J-L,N-P) at levels indicated by dashed lines in A,E,I,M. *Vangl1* mRNA expression is confined to midline neuroepithelium, from hindbrain to low trunk (arrows), in E8.5 wild-type embryos (*+/+*; A-D). There is no ectopic expression in *Vangl2^Lp/Lp^* embryos (*Lp/Lp*; E-H) nor overlap with *Erbb3*-positive NC (I-L). *Vangl2* is expressed throughout the neuroepithelium (M-P), overlapping with *Vangl1* only in midline cells, and not overlapping with *Erbb3*. Scale bars: 200 µm.

*Vangl1* expression was detected solely in the ventral neuroepithelium of E8.5 *Vangl2^+/+^* and *Vangl2^Lp/Lp^* embryos, from the level of hindbrain to low spine (Fig. 2A,E). In both genotypes, *Vangl1* transcripts could not be detected in the upper hindbrain, midbrain (Fig. 2B,F) or edges of the trunk neural folds (Fig. 2C,D,G,H), which are all sites of *Erbb3*-positive NC cell origin (Fig. 2I-L). *Vangl2* expression also showed no overlap with *Erbb3*, but rather exhibited generalised neural tube expression, overlapping with *Vangl1* only at the ventral midline (Fig. 2M-P). Later in neurulation, *Vangl1* expression remained distinct from *Erbb3* along the body axis, with no evidence of ectopic expression in *Vangl2^Lp/Lp^* embryos (supplementary material Fig. S3).

**Fig. 3.**
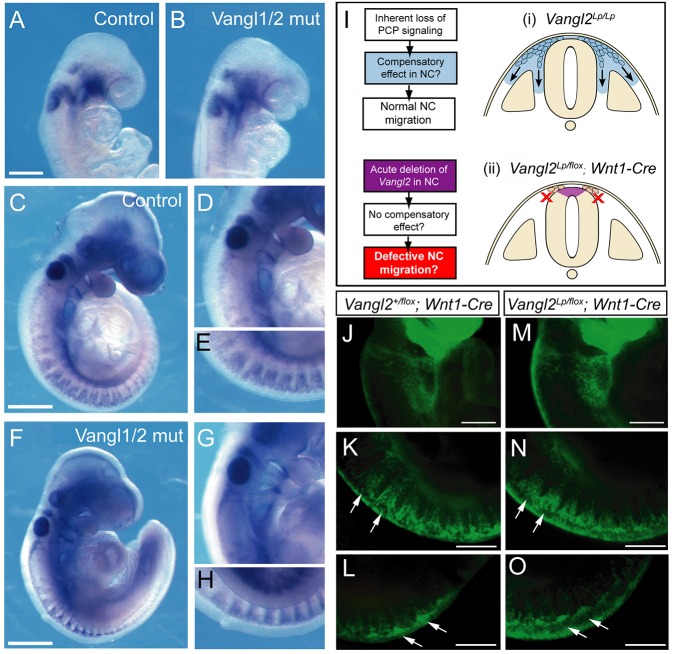
**Normal NC migration in *Vangl1/2* double mutants and after acute *Vangl2* downregulation in the NC lineage.** (A-H) Control (A,C-E; *Vangl1^gt/+^; Vangl2^Δ/+^*) and double-mutant (B,F-H; *Vangl1^gt/gt^; Vangl2^Δ/Δ^*) embryos exhibit normal migration of *Erbb3*-positive cranial NC (E8.5; A,B) and cranial/trunk NC (E9.5; C-H). Acute NC downregulation of *Vangl2* to test for a possible compensatory mechanism in *Vangl2^Lp/Lp^* embryos (I) reveals identical YFP-positive NC migration in control (J-L; *Vangl2^+/flox^; Wnt1-Cre*) and downregulation (M-O; *Vangl2^Lp/flox^; Wnt1-Cre*) E9.5 embryos. Arrows indicate comparable streams of NC cells migrating from the trunk neural tube in both genotypes. Scale bars: 200 µm in A; 500 µm in C,F; 100 µm in J-O.

To test experimentally whether *Vangl1* may compensate for *Vangl2* disruption in NC migration, we bred mice doubly homozygous for *Vangl1* and *Vangl2* loss of function (Song et al., 2010). The pattern of *Erbb3*-positive NC cell migration was very similar at both E8.5 and E9.5 in normally developing controls (*Vangl1^gt/+^;* *Vangl2^Δ/+^*; Fig. 3A,C-E) and in doubly homozygous mutants (*Vangl1^gt/gt^;* *Vangl2^Δ/Δ^*; Fig. 3B,F-H), despite the entirely open neural tube in the latter embryos. We conclude that *Vangl* gene function is not required for mouse NC migration *in vivo*.

### Acute ablation of Vangl2 function in the NC lineage

Constitutional absence of Vangl2-dependent PCP signalling in *loop-tail* embryos could stimulate a compensatory mechanism (e.g. activation of a Vangl2-independent pathway) in the NC or surrounding tissue, allowing normal NC migration (Fig. 3I, i). To address this, we produced *Vangl2^Lp/flox^; Wnt1-Cre/YFP* embryos in which *Vangl2* expression was ablated specifically in the NC lineage. We reasoned that acute ablation of *Vangl2* should prevent any compensatory mechanism from arising, and so lead to NC migration defects (Fig. 3I, ii). Fluorescently labelled NC cells were detected in E9.5 *Vangl2^Lp/flox^; Wnt1-Cre/YFP* embryos in a pattern indistinguishable from that of controls (Fig. 3J-O). WISH for *Erbb3* revealed no difference between conditional mutants and controls (data not shown). We conclude that the normal pattern of NC migration observed in *Vangl2^Lp/Lp^* embryos is unlikely to arise from a compensatory mechanism masking a role for Vangl2 in NC migration.

### *Vangl2^Lp/Lp^* NC cells migrate normally *in vitro*

**Fig. 4.**
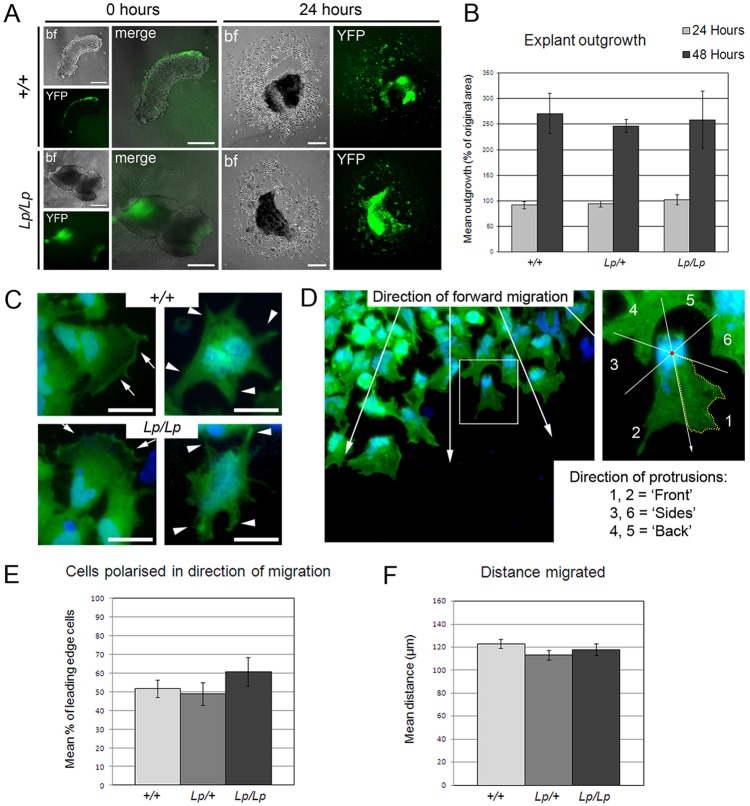
**NC cells migrate similarly from *Vangl2^+/+^* and *Vangl2^Lp/Lp^* explant cultures.** YFP-positive NC are initially (0 h; A) on the dorsal margin of *Vangl2^+/+^; Wnt1-Cre/YFP* (*+/+*) explants and on *Vangl2^Lp/Lp^; Wnt1-Cre/YFP* (*Lp/Lp*) neural fold tips. Cells emerge in similar numbers (at 24 h; A), with no difference in outgrowth area (*P*=0.91, one-way ANOVA; B). Leading edge NC cells (anti-GFP/YFP; DAPI) are polarised (C, arrows) or non-polarised (C, arrowheads). Analysis of leading edge cells (D) reveals no difference between genotypes in the proportion of polarised cells nor in the mean distance migrated (*P*=0.42, E; *P*=0.21, F; one-way ANOVA). bf, bright field. Error bars indicate s.e.m. At least three explants were studied per genotype and time point. Scale bars: 200 µm in A; 50 µm in C.

Migration of *Xenopus* NC was inhibited after disruption of PCP signalling (De Calisto et al., 2005). By contrast, we observed comparable *in vitro* outgrowth of migratory cells from *Vangl2^+/+^* and *Vangl2^Lp/Lp^* neural tube explants (supplementary material Fig. S4A,B). YFP-positive premigratory NC cells were initially detected along the dorsal margin of neural tube/fold explants from *Vangl2^+/+^* and *Vangl2^Lp/Lp^* embryos expressing *Wnt1-Cre/R26R-YFP*. After 24 h, similar numbers of YFP-positive migratory cells had emerged from the explants of both genotypes (Fig. 4A). The percentage increase in outgrowth area did not differ between *Vangl2^+/+^*, *Vangl2^Lp/+^* and *Vangl2^Lp/Lp^* genotypes at either 24 or 48 h (Fig. 4B). Double immunostaining confirmed that the majority of YFP-positive NC cells also expressed the NC cell marker p75 (Ngfr – Mouse Genome Informatics) (supplementary material Fig. S4D,E).

In *Xenopus* NC outgrowths, leading edge cells extended large, polarised lamellipodia whereas those with defective PCP signalling failed to polarise (Carmona-Fontaine et al., 2008; De Calisto et al., 2005). In mouse *Vangl2^+/+^* and *Vangl2^Lp/Lp^* explants, we observed both highly polarised YFP-expressing cells at the leading edge as well as non-polarised cells (Fig. 4C; supplementary material Fig. S4C). The proportion of cells polarised in the direction of migration did not differ significantly between genotypes (Fig. 4D,E), nor did the distance migrated by leading edge NC cells from the central explant (Fig. 4F). Together, these data demonstrate that loss of function of the core PCP gene *Vangl2* does not impair NC cell migration *in vitro*.

## DISCUSSION

In contrast to *Xenopus* and zebrafish, where Wnt/PCP signalling is required for NC migration (Carmona-Fontaine et al., 2008; Matthews et al., 2008), we could detect no abnormality of NC development in *Vangl1/2* mouse mutants with severe PCP defects. NC migration disorders are typically associated with anomalies of craniofacial development and cardiac outflow tract (OFT) septation, but neither defect is observed in *Vangl2^Lp/Lp^* fetuses (Henderson et al., 2001). Pairwise loss of mouse dishevelled genes *Dvl1/2* and *Dvl2/3* does cause cardiac OFT defects but cardiac NC migration has been described in these mice as either normal (Etheridge et al., 2008) or disrupted via a disorder of Wnt/β-catenin signalling (Hamblet et al., 2002). Canonical Wnt/β-catenin signalling is known to be required for NC migration in mice (Ikeya et al., 1997). We conclude that the PCP dependence of NC development is not universal among vertebrates.

Several lines of evidence indicate that PCP signalling is abrogated in *Vangl2^Lp/Lp^* mice. Vangl2 recruits all three dishevelled family members to the plasma membrane (Torban et al., 2004) as part of the asymmetric localisation of PCP protein complexes needed for signal transduction. Membrane localisation is lost in *Vangl2^Lp/Lp^* embryos (Torban et al., 2007). Moreover, the *Vangl2^Lp^* allele acts as a dominant negative in the female reproductive tract and brain ependymal cells (Guirao et al., 2010; vandenBerg and Sassoon, 2009). Stronger neural tube and inner ear phenotypes occur in *loop-tail* mice than in *Vangl2* knockouts, supporting a dominant-negative effect of the *Vangl2^Lp^* allele (Song et al., 2010; Yin et al., 2012). This is likely to result from disrupted trafficking from endoplasmic reticulum to plasma membrane, which affects the Vangl2^Lp^ protein (Merte et al., 2010) and other PCP proteins in *Vangl2^Lp/Lp^* mice (Yin et al., 2012).

We could not detect functional redundancy between *Vangl1* and *Vangl2* in relation to NC migration. Moreover, acute downregulation of *Vangl2* in the NC lineage did not suggest a compensatory mechanism in mice with constitutional lack of Vangl2. *Vangl2* is expressed at the mRNA level in the mouse neural tube but not in migrating NC cells. Similarly, mRNAs for other core PCP components, including *Celsr1* (Formstone and Little, 2001; Shima et al., 2002) and *Dvl1* (Gray et al., 2009), are not detected in NC. Hence, our finding of normal NC migration in *loop-tail* mice is consistent with the absence of PCP signalling in NC cells after emigration from the neural tube.

Neurocristopathies are congenital malformations involving defective NC development (Bolande, 1974). These include craniofacial anomalies, gut innervation defects and disorders of cardiac OFT septation, which occur in ∼5 per 1000 births (Dolk et al., 2010). Environmental factors (e.g. alcohol, retinoic acid) are relatively minor causes of birth defects [0.12 cases per 1000 births (Dolk et al., 2010)] and genetic factors are likely to be quantitatively more significant. Core PCP genes have been implicated in human neural tube closure defects (Juriloff and Harris, 2012) and, given the close spatiotemporal relationship between neurulation and NC development, PCP genes might be considered as strong candidates for congenital neurocristopathies. Our findings, however, argue that PCP genes are an unlikely cause of NC-related birth defects. Instead, attention should be focused on other groups of genes, such as those regulating the guidance of migrating NC cells and the differentiation of NC derivatives.

## MATERIALS AND METHODS

### Mouse strains and embryos

Animal studies were performed according to the UK Animals (Scientific Procedures) Act 1986 and the Medical Research Council's Responsibility in the Use of Animals for Medical Research (July 1993). Experimental embryos were generated from strains: *Vangl2^Lp/+^* (CBA/Ca background) (Ybot-Gonzalez et al., 2007), *Wnt1*-*Cre* (Jiang et al., 2000) crossed with *R26R-EYFP* (Srinivas et al., 2001), doubly heterozygous *Vangl1^gt/+^;* *Vangl2^Δ/+^* mice (Song et al., 2010) and *Vangl2^flox/flox^* (gift from Deborah Henderson, Institute of Genetic Medicine, Newcastle University, UK). See supplementary material methods for breeding schemes and genotyping. Noon after overnight mating was designated E0.5. Embryos were dissected at E8.5-10.5 in Dulbecco's Modified Eagle's Medium (DMEM) containing 10% fetal calf serum (FCS). Whole-mount YFP expression was visualised by direct fluorescence. Embryos for WISH or immunohistochemistry were fixed in 4% paraformaldehyde (PFA) in PBS at 4°C overnight.

### WISH, immunohistochemistry and immunocytochemistry

WISH was performed on a minimum of five embryos per probe and per genotype. Digoxygenin-labelled RNA probes for *Erbb3*, *Vangl1* and *Vangl2* were as described (Doudney et al., 2005; Henderson et al., 2001). Hybridised embryos were embedded in 2% agarose in PBS and vibratome-sectioned at 50 µm thickness before mounting in Mowiol (Sigma). Immunohistochemistry utilised 7 µm wax sections; primary and secondary antibodies are listed in supplementary material methods. Sections and explants were mounted using Vectashield medium with DAPI (Vector Labs).

### Neural tube explant culture

Embryo trunks were digested in 2% pancreatin (Sigma) in PBS at 37°C for 15 min, and the neural tube adjacent to the caudalmost five somites was plated onto fibronectin- and poly-D lysine-coated cover-glasses. Explants were cultured at 37°C (5% CO_2_/95% air): from 0-24 h in Phenol Red-free DMEM containing 10% FCS plus 1% penicillin and streptomycin; and from 24-48 h in serum-free DMEM containing supplements for neural cell culture (N2 and B27; Invitrogen) and growth factors (EGF and FGF). After culture, explants were fixed for 10 min in 4% PFA. See supplementary material methods for details of NC cell counts and migration analysis.

### Statistical analysis

Statistical tests were performed using SigmaStat (Systat) version 3.5.

## Supplementary Material

Supplementary Material
