# Supplementary material for: Vangl-dependent planar cell polarity signalling is not required for neural crest migration in mammals
Source: Development. 2014 Aug;141(16):3153–8. doi: 10.1242/dev.111427 (PMC4197537; doi:10.1242/dev.111427)
Supplement: Supplementary Material [file supp_141_16_3153__index.html]

Vangl-dependent planar cell polarity signalling is not required for neural crest migration in mammals — Supplementary Material 

# Vangl-dependent planar cell polarity signalling is not required for neural crest migration in mammals

## DEV111427 Supplementary Material

**Files in this Data Supplement:**

- **Supplementary Material**
