# Supplementary material for: Vangl-dependent planar cell polarity signalling is not required for neural crest migration in mammals
Source: Development. 2014 Aug;141(16):3153–8. doi: 10.1242/dev.111427 (PMC4197537; doi:10.1242/dev.111427)
Supplement: Supplementary Material [file supp_141.16.3153_DEV111427.pdf]

SUPPLEMENTARY METHODS

Mouse breeding and genotyping

Mice heterozygous for the *loop-tail* mutation (*Vangl2*$^{Lp/+}$; CBA/Ca background) were intercrossed to generate wild-type (*Vangl2*$^{+/+}$) and homozygous mutant (*Vangl2*$^{Lp/Lp}$) embryos. *Vangl2*^{Lp} genotyping was performed using the *Crp* microsatellite DNA marker, as described (Copp et al., 1994).

To generate embryos in which the NC cells and descendants were fluorescently labelled, crosses were performed between *Wnt1-Cre* (Jiang et al., 2000) and *R26R-EYFP* reporter (Srinivas et al., 2001) mice. *Wnt1-Cre; YFP* offspring were bred with *Vangl2*$^{Lp/+}$ mice and the *Vangl2*$^{Lp/+}$*; Wnt1-Cre/YFP* offspring were intercrossed to generate *Vangl2*$^{+/+}$*; Wnt1-Cre/YFP*

and *Vangl2*$^{Lp/Lp}$*; Wnt1-Cre/YFP* embryos for analysis.

Doubly homozygous *Vangl1*$^{gt/gt}$*; Vangl2*$^{\Delta/\Delta}$ embryos were produced by matings between doubly heterozygous *Vangl1*$^{gt/+}$*; Vangl2*$^{\Delta/+}$ mice, which were bred and genotyped as described previously (Song et al., 2010).

To ablate *Vangl2* specifically in the NC lineage, *Vangl2*$^{Lp/+}$*; Wnt1-Cre/YFP* mice were crossed with *Vangl2*$^{flox/flox}$ animals (gift of Prof. Deborah Henderson). The experimental offspring were *Vangl2*$^{Lp/flox}$*; Wnt1-Cre* or *Vangl2*$^{Lp/flox}$*; Wnt1-Cre/YFP* (i.e. *Vangl2*$^{Lp/–}$ in the Wnt1-positive NC cells, but *Vangl2*$^{Lp/+}$ in all other tissues). Control offspring were *Vangl2*$^{+/flox}$*; Wnt1-Cre* (or *Vangl2*$^{+/flox}$*; Wnt1-Cre/YFP*), *Vangl2*$^{+/flox}$ and *Vangl2*$^{Lp/flox}$.

NC cell counts

Sections from *Vangl2*$^{+/+}$*; Wnt1-Cre/YFP* and *Vangl2*$^{Lp/Lp}$*; Wnt1-Cre/YFP* embryos were analysed at the level of the developing heart (*n*= at least 4 sections per genotype), trunk (*n*=3 sections per genotype) and foregut/trachea (*n*=5 sections per genotype). Sections were matched for body level between genotypes and total migrating YFP-labelled NC cells were counted in each section.

Analysis of NC cell migration *in vitro*

The rate of cell migration over 2 days was assessed by measuring the area of outgrowth (outer dotted lines in Fig. S4B) as a percentage increase of the area covered by the central

explant tissue at 24 and 48 h (inner dotted lines; at least three samples measured per genotype and time point). This allowed for any variation due to differences in the original size of the explant, or to pieces of the tissue detaching and floating away during the first few hours of culture. Migration rate was analysed by measuring the distance travelled by YFP-positive leading edge NC cells from the periphery of the central explant tissue. Polarity of NC cells at the leading edge was assessed by measuring the distribution of cell area relative to the direction of migration, with 'forward' defined as the direction away from the central mass of neuroepithelial tissue. The cell area within six circular sectors, centred on the nucleus, was measured, allowing each cell to have designated 'front', 'sides' and 'back' based on the direction in which the majority of the area was distributed (Fig. 4D). Cells that did not display a majority of at least 5% in any direction were designated 'none'. Cells which were polarised in the direction of forward migration were defined as those with the majority of their area distributed towards the 'front'. Migration rate and polarity analysis were performed on at least 64 cells from three explants for each genotype. All area and distance measurements were performed using ImageJ software.

Antibodies

Primary antibodies: Chicken polyclonal anti-GFP/YFP (Abcam, ab13970; dilutions 1:100 for sections; 1:250 for explant cultures); rabbit polyclonal anti-P75 (Santa Cruz, sc-8317; dilution 1:200). Secondary antibodies: Fluorescein-labelled goat anti-chicken IgY (Aves Labs, F-1005; dilution 1:200); goat anti-chicken IgG-Alexa488 (Invitrogen, A11039; dilution 1:400; goat anti-rabbit IgG-Alexa568 (Invitrogen, A21069; dilution 1:400).

Supplementary references

Copp, A.J., Checiu, I. and Henson, J.N. (1994). Developmental basis of severe neural tube defects in the *loop-tail* (*Lp*) mutant mouse: use of microsatellite DNA markers to identify embryonic genotype. *Dev. Biol.* **165**, 20-29.

Jiang, X.B., Rowitch, D.H., Soriano, P., McMahon, A.P. and Sucov, H.M. (2000). Fate of the mammalian cardiac neural crest. *Development* **127**, 1607-1616.

Song, H., Hu, J., Chen, W., Elliott, G., Andre, P., Gao, B. and Yang, Y. (2010). Planar cell

polarity breaks bilateral symmetry by controlling ciliary positioning. *Nature* **466**, 378-382.
Srinivas, S., Watanabe, T., Lin, C.S., William, C.M., Tanabe, Y., Jessell, T.M. and Costantini, F. (2001). Cre reporter strains produced by targeted insertion of EYFP and ECFP into the ROSA26 locus. *BMC Dev. Biol.* **1**, 4.

Pryor et al: Supplementary Figures

Figure S1

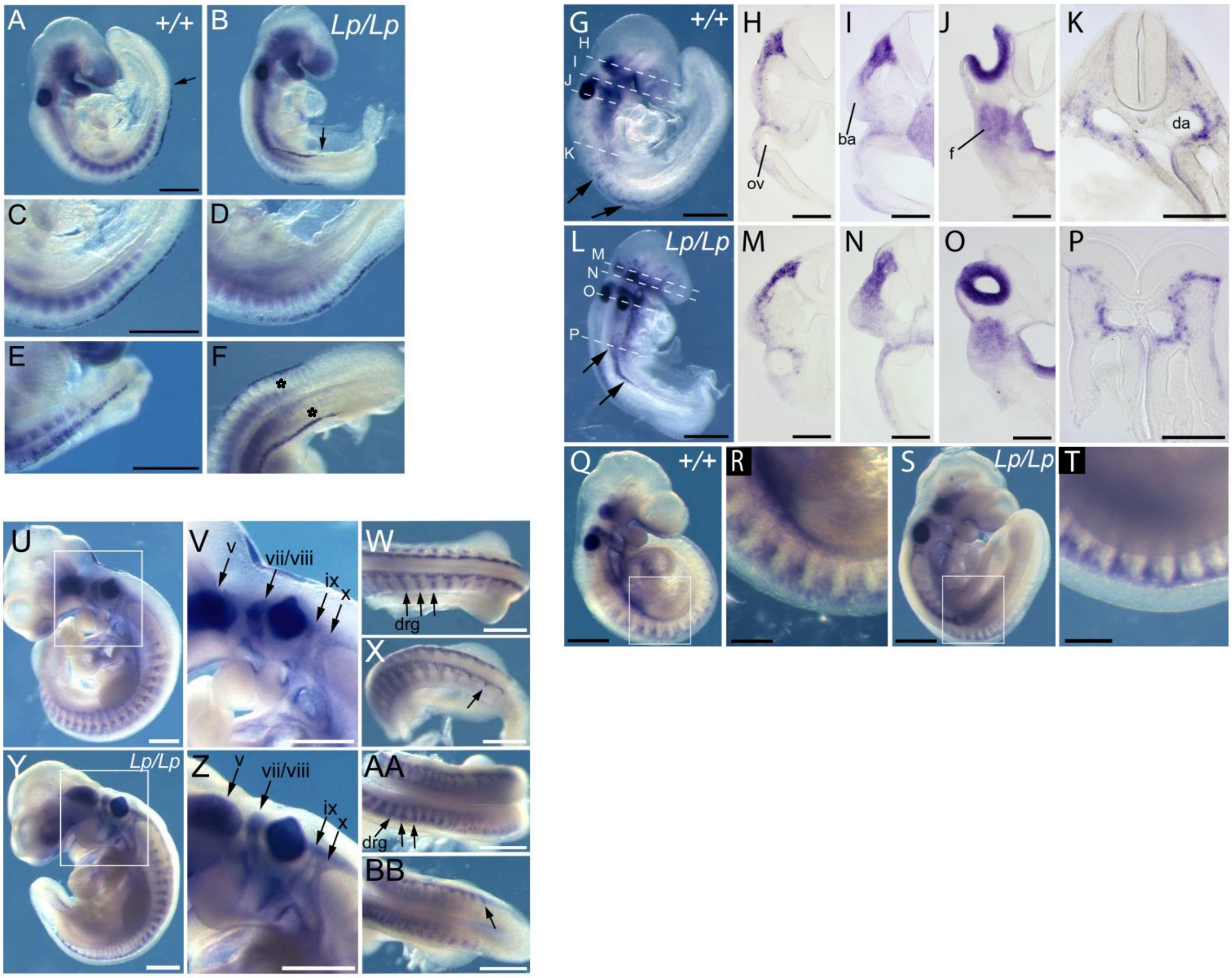

NC specification, migration and derivative formation appear normal in *Vangl2*$^{Lp/Lp}$ embryos.

Whole-mount in situ hybridization for pre-migratory NC marker *Sox9* (A-F) and migratory NC marker *Erbb3* (G-BB) in wild-type (+/+; A, C, E, G-K, Q, R, U-X) and *Vangl2*$^{Lp/Lp}$ (*Lp/Lp*; B, D, F, L-P, S, T, Y-BB) embryos.

(A-F) *Sox9* labels pre-migratory E9.5 NC cells as far caudally as recently formed somites (arrows in A, B), with no difference between genotypes. NC cells are specified on open neural fold tips in *Lp/Lp* (* in F).

(G-P) *Erbb3* marks a rostral-caudal progression of E9.5 trunk NC cells emigrating from the neural tube (arrows in G, L). Sections confirm closely similar NC migration patterns in both genotypes (H-K and M-P).

(Q-T) Streams of NC cells migrate similarly in the trunk of E10.5 *+/+* and *Lp/Lp* embryos.

(U-BB) *Erbb3* expression in E10.5 NC-derived trigeminal (v), facio-acoustic (vii/viii), glossopharyngeal (ix), and vagal (x) cranial ganglia (U, V, Y, Z) and in dorsal root ganglia (drg; W, AA) of *+/+* and *Lp/Lp* embryos. Emerging NC are visible in low trunk region of both genotypes (arrows in X, BB)

Abbreviations: ba, branchial arch; da, dorsal aorta; f, facial NC; ov, optic vesicle.

Scale bars: 500 µm (A-F, G, L, Q, S, U-BB); 200 µm (H-K, M-P, R, T).

Figure S2

Fluorescent labelling of NC reveals normal migration and derivative formation in *Vangl2*$^{Lp/Lp}$ embryos.

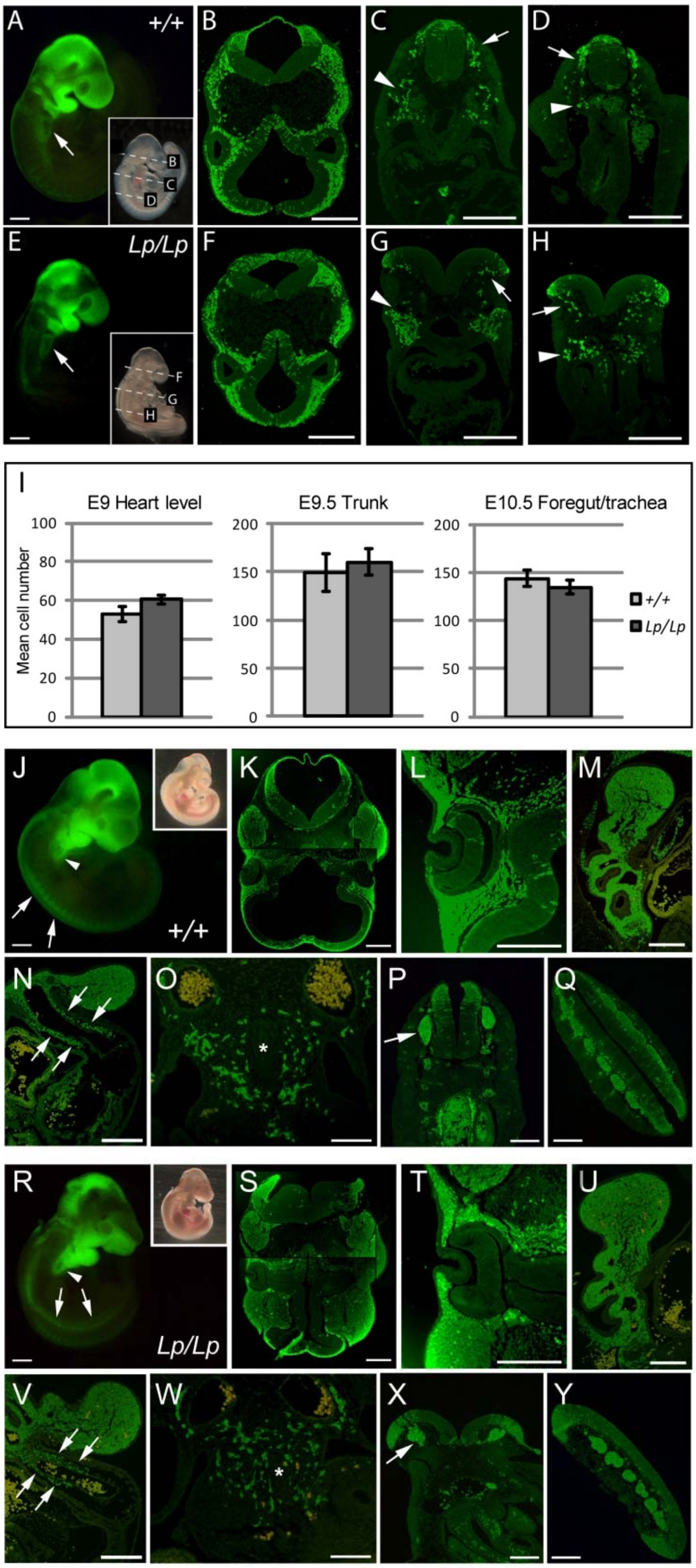

NC and its YFP-expressing descendants in *Vangl2*$^{+/+}$*; Wnt1-Cre/YFP* (*+/+*; A-D, J-Q) and *Vangl2*$^{Lp/Lp}$*; Wnt1-Cre/YFP* (*Lp/Lp*; E-H, R-Y) embryos.

(A-H) E9.5 embryos showing NC colonization, similarly in *+/+* and *Lp/Lp*, of upper and lower branchial arches (arrows: A, E), and forebrain/hindbrain (B, F). NC cells are emigrating from neural tube (arrows: C, D, G, H) and colonizing regions lateral to foregut and around paired aortae (arrowheads: C, D, G, H).

(I) Number of migrating NC cells (mean ± SEM) does not differ between genotypes for each region analysed (heart level, p = 0.183; trunk, p = 0.446; foregut/trachea, p = 0.664).

(J-Y) E10.5 embryos showing YFP-positive NC cells within the nasal process, branchial arches, branchial pouches (arrowheads in J, R) and dorsal root ganglia (arrows in J, R). Despite the widely open neural folds in *Lp/Lp*, no differences in distribution of YFP-expressing NC cells are observed between genotypes in sections. NC derivatives are detected sub-epidermally (K, S), around the developing eye (L, T), and in the branchial pouches (M, U). NC cells are migrating into the developing heart (arrows in N, V) and around the foregut (asterisks in O, W). YFP-positive dorsal root ganglia are normally sized in *Lp/Lp* (arrows in P, X, also Q, Y). Auto-fluorescent blood cells appear yellow.

Scale bars: 100 μm (A-H, K-Q, S-Y), 500 μm (J, R).

Figure S3

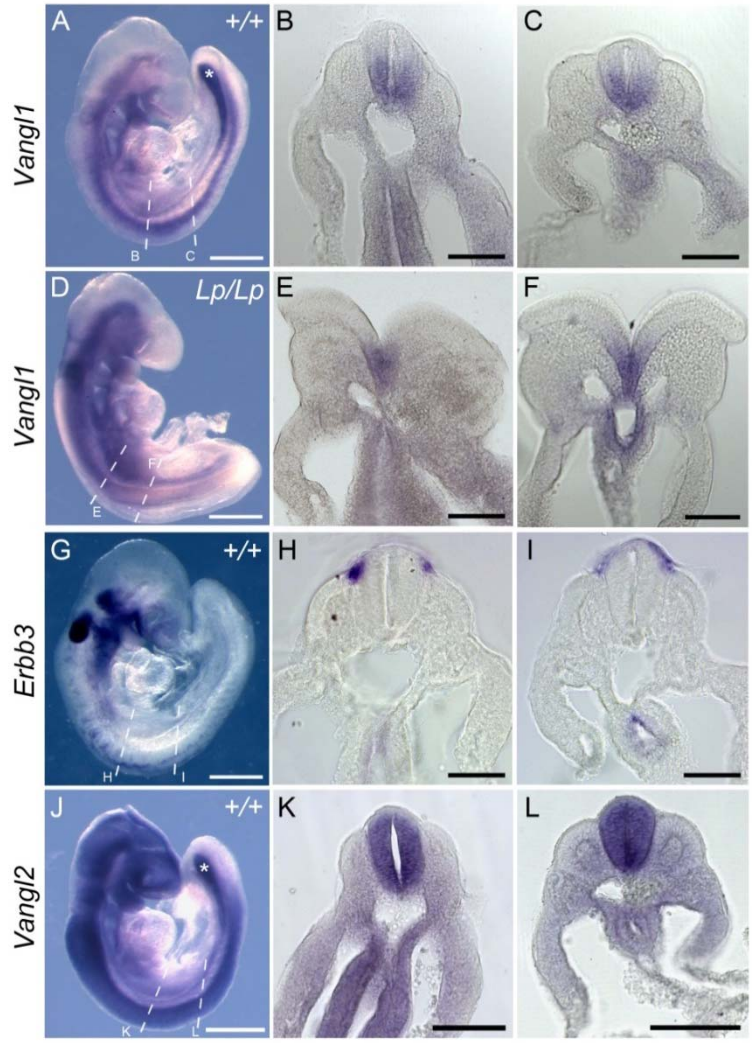

***Vangl1* is not expressed in wild-type trunk NC, nor ectopically expressed in *Vangl2*$^{Lp/Lp}$ mutants.**

(A-C) Whole-mount in situ hybridisation for *Vangl1* in wild-type (WT; *+/+*) embryo at E9.5. Transcripts are detected in the ventral half of the neural tube from midbrain to posterior neuropore (* in A indicates probe trapped within the hindgut). This expression pattern is confirmed in transverse sections (B, C; at level indicated by dotted lines in A).

(D-F) Pattern of *Vangl1* expression in *Vangl2*$^{Lp/Lp}$ appears unaltered compared with WT. Marginally more intense cranial staining in *Vangl2*$^{Lp/Lp}$ (*Lp/Lp*; D), is likely an artefact due to the widely open neural folds. Transverse sections (E-F) reveal *Vangl1* expression confined to the ventral neuroepithelium at a similar intensity as WT.

(G-I) *Erbb3* mRNA expression is confined to migrated NC in the cranio-facial region (G) and actively migrating NC cells in the trunk (H, I). *Vangl1* and *Erbb3* expression does not overlap in either genotype.

(J-L) *Vangl2* mRNA expression at E9.5 is present at greatest intensity throughout the neuroepithelium, and at lower intensity in mesoderm and gut endoderm (* in J indicates probe trapped within the hindgut). As at E8.5, co-expression of *Vangl1* and *Vangl2* occurs only in ventral midline neural tube cells, and Vangl2 does not overlap in expression with *Erbb3*.

Scale bars: 500 µm (A, D, G, J), 200 µm (all sections).

Figure S4

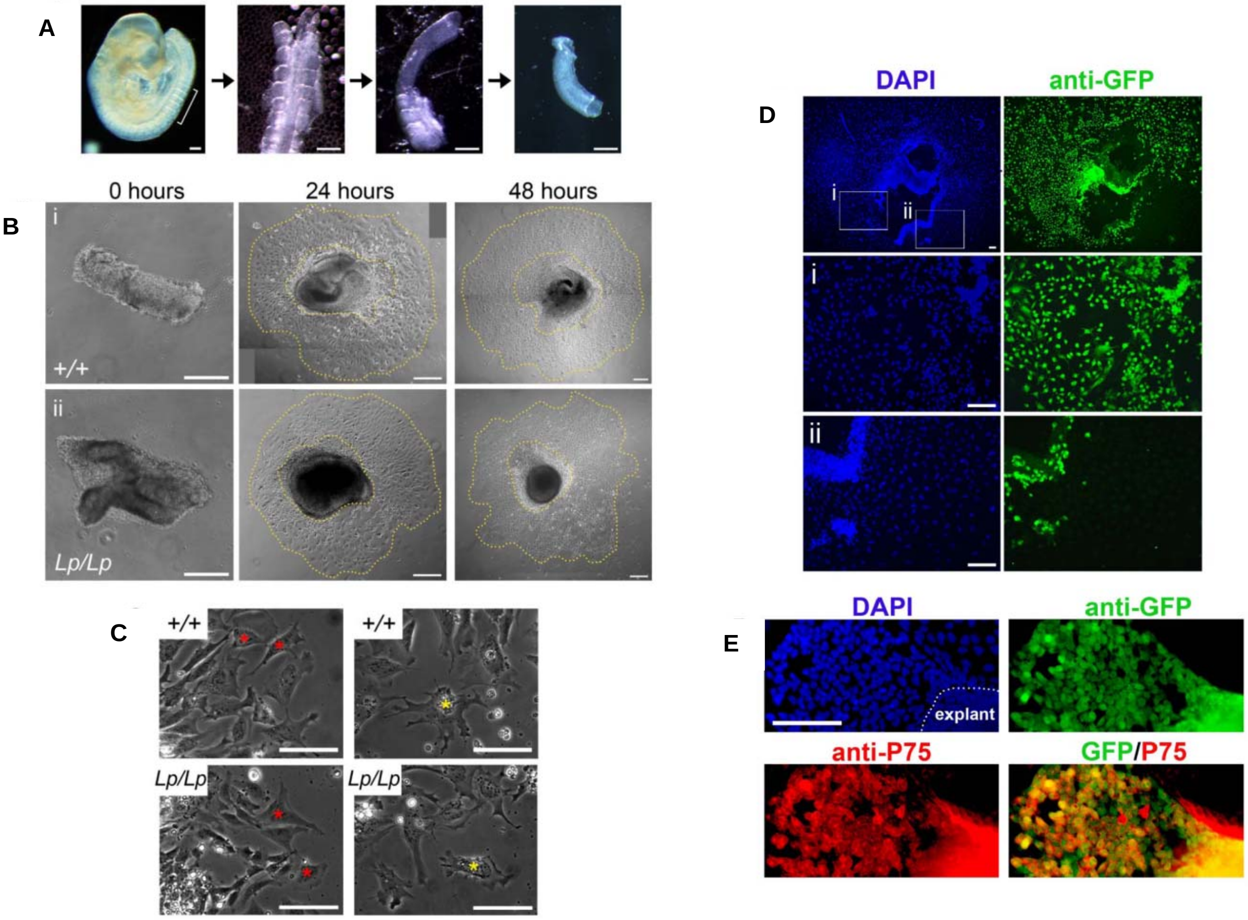

Neural crest outgrowth *in vitro* is equivalent in wild-type and *Vangl2*$^{Lp/Lp}$ explants.

(A) Method of preparation of neural tube explants for outgrowth culture. After enzymatic digestion, the neural tube was isolated adjacent to the posterior-most five somites (indicated by white line in left panel). Surrounding surface ectoderm and somitic tissues were removed and explants were plated for culture on fibronectin/ poly-D lysine-coated coverslips.

(B) Wild-type (*+/+*; i) and *Vangl2*$^{Lp/Lp}$ (*Lp/Lp*; ii) neural tube explants cultured for 24 and 48 h. Inner yellow dotted lines indicate the area covered by the central mass of neuroepithelial tissue; outer dotted lines indicate the leading edge of the migratory population.

(C) Examples of the variable morphology of leading edge cells, as viewed by phase contrast microscopy. Red asterisks: wild-type and *Vangl2*$^{Lp/Lp}$ cells which appear polarised. Yellow asterisks: cells which extend protrusions in all directions.

(D) Representative *Vangl2*$^{+/+}$*; Wnt1-Cre/YFP* neural tube explant culture immunostained with anti-GFP. Higher magnification views of the boxed regions (i and ii) are shown in the lower panels. While the majority of migratory cells are YFP-positive NC cells (i), other cell types, which do not express YFP, are also present (ii).

(E) Representative *Vangl2*$^{+/+}$*; Wnt1-Cre/YFP* neural tube explant culture immunostained with anti-GFP and anti-P75. Most YFP-positive cells also express the NC marker P75 *in vitro*.

Scale bars: 200 µm (A, B), 50 µm (C), 100 µm (D, E).
